# Development and Implementation of an Online Patient Education Program for Children and Adolescents With Myalgic Encephalomyelitis/Chronic Fatigue Syndrome, Their Parents, Siblings, and School Personnel: Protocol for the Prospective BAYNET FOR ME/CFS Study

**DOI:** 10.2196/54679

**Published:** 2024-11-21

**Authors:** Franca Keicher, Julia Thomann, Jana Erlenwein, Mara Schottdorf, Nils Lennart Reiter, Nadine Patricia Scholz-Schwärzler, Barbara Vogel, Cordula Warlitz, Silvia Stojanov, Silvia Augustin, Lola Goldbrunner, Linda Schanz, Veronika Dodel, Charlotte Zipper, Nicole Schiweck, Robert Jaeschke, Milica Saramandic, Karolina Wiejaczka, Maria Eberhartinger, Kristina Dettmer, Daniel Bruno Ricardo Hattesohl, Stephanie Englbrecht, Uta Behrends, Juliane Spiegler

**Affiliations:** 1 Department of Child and Adolescent Psychiatry, Psychosomatics and Psychotherapy University Hospital of Wuerzburg Würzburg Germany; 2 Department of Pediatrics University Hospital of Wuerzburg Würzburg Germany; 3 PhysioBib GbR Berlin Germany; 4 MRI Chronic Fatigue Center for Young People Center for Pediatric and Adolescent Medicine Technical University of Munich Munich Germany; 5 Department of Orthopedics and Sports Orthopedics, Physical Therapy University Hospital rechts der Isar Technical University of Munich Munich Germany; 6 Rehabilitation Centre for Children With Respiratory Diseases Fachkliniken Wangen Wangen Germany; 7 Parent Initiative for Children and Adolescents with ME/CFS Munich e.V. Munich Germany; 8 German Association for ME/CFS e. V. Hamburg Germany

**Keywords:** patient education, ME/CFS, children, adolescents, ModuS, parents, teachers, siblings, training

## Abstract

**Background:**

Myalgic encephalomyelitis/chronic fatigue syndrome (ME/CFS) presents significant challenges for affected children and adolescents, their social environment, and treating physicians, due to its profound impact on quality of life and the lack of causal therapeutic approaches. One crucial aspect of care that has been missing for these patients is comprehensive education for both them and their social circles.

**Objective:**

This study protocol aims to outline the goals, study design, execution, and evaluation of the subproject within the BAYNET FOR ME/CFS project. The focus is on developing online education programs for children and adolescents with ME/CFS, as well as for their parents, siblings, and school staff. These programs are designed to improve independent disease management, increase knowledge, and promote interaction with other affected individuals.

**Methods:**

In phase I, the group-based online education programs were developed by a multidisciplinary team based on the ModuS concept created by the Competence Network for Patient Education (KomPaS). These programs were then piloted and finalized. Phase II involved recruiting participants and implementing the finalized programs. Given the restricted physical and cognitive capacities of the affected individuals, the patient education programs were exclusively designed in a digital format to facilitate participation. In phase III, the programs will be evaluated for acceptance, completeness, and participant satisfaction. The qualitative assessment will focus on individual expectations and benefits derived from the training. Phase IV will further assess the programs in terms of improvements in disease knowledge, health-related quality of life, life satisfaction, and family burden.

**Results:**

The programs were developed, piloted, and finalized during phase I, which ran from December 2022 to May 2023. The pilot phase, from March to May 2023, led to adaptations in the program concept. In total, 8 patients and their parents, 5 siblings, and 59 school staff participated in the piloting. Adjustments were made to the format, content, duration, and schedule to better meet the needs of the affected individuals and their social circles. In phase II, participant recruitment for the patient education program took place from January to July 2023. The study successfully recruited 24 young patients with ME/CFS and their parents, along with 8 siblings and 51 school staff. Two program blocks for patients and parents and 2-3 blocks for siblings and school staff commenced in May 2023 and were completed within the same year. Phase III began after phase II and involves the evaluation of the programs, with the process expected to conclude by the end of 2024. Phase IV, planned for 2025-2026, will involve the rollout of the program to 150 children and their caretakers. This phase will focus on evaluating disease knowledge, health-related quality of life, life satisfaction, and family burden, as well as include longitudinal assessments.

**Conclusions:**

The data aim to support the development of a comprehensive, interprofessional care model for children and adolescents with ME/CFS.

**International Registered Report Identifier (IRRID):**

DERR1-10.2196/54679

## Introduction

### Background

Myalgic encephalomyelitis/chronic fatigue syndrome (ME/CFS) is a severe chronic illness that causes significant life changes for those affected as well as their social environment [1,2]. It is estimated that approximately 300,000 people in Germany suffer from ME/CFS, including up to 90,000 children and adolescents [3]. An increase in cases has been observed during the COVID-19 pandemic [4]. Given these numbers, ME/CFS is not considered a rare disorder [5]. Despite ongoing research, the underlying pathophysiology of ME/CFS remains only partially understood [6]. Infections are often identified as the most common triggers [7]. The primary symptoms include profound fatigue that does not improve with rest and can lead to limitations in all areas of school, leisure, and social activities [8]. Another diagnostic criterion is postexertional malaise (PEM), characterized by a decrease in functional level or exacerbation of symptoms after minimal physical, cognitive, or emotional effort, often described as a “crash.” This often occurs with a time delay after exertion and can last from hours to months [8,9]. Additionally, various forms of muscle pain, joint pain, and headaches are reported, along with sleep disturbances and orthostatic intolerances, which can manifest as postural orthostatic tachycardia syndrome [6,8,10,11]. The disease significantly reduces health-related quality of life (HRQoL) [12]. This reduction is attributed not only to the symptoms themselves but also to their impact on school or occupational participation, often resulting in reduced attendance or complete absence from school. Consequently, social isolation frequently occurs [13].

To address knowledge deficits and empower individuals with ME/CFS to engage in self-management and disease control, experts from the European Network on ME/CFS (EUROMENE), which defines diagnostic standards for adults, recommend patient education as part of the therapeutic measures [14]. Patient education programs have become an integral part of therapy options for children and adolescents with various chronic diseases. The objective of these programs is to promote self-management among individuals and within their social environment. Several studies have demonstrated that patient education programs lead to increased self-efficacy, disease competence, and satisfaction [15,16], as well as improved HRQoL and a reduction in disease-specific burden [17]. Additionally, disease-specific symptoms decreased, and the risk of relapses or exacerbations was reduced [18,19]. One established and validated concept is the standardized modular training program, ModuS, developed by the Competence Network for Patient Education (KomPaS) with support from the Federal Ministry of Health [20]. ModuS is available for some less common and rare chronic illnesses for individuals [18,20], as well as for siblings [21] and parents. The training concept consists of 4 generic modules and 3 disease-specific components [20]. The generic modules address psychosocial topics relevant to all children and adolescents with chronic diseases. By contrast, the disease-specific modules provide detailed information about the etiology, course, and therapeutic measures for the respective condition. The structure of the ModuS training allows it to be adapted to various chronic diseases [20]. Additionally, these programs can be customized to meet the needs of other groups, such as parents, teachers, and siblings [20,21].

### Study Goal

Currently, there is no standardized education program for children and adolescents with ME/CFS and their surroundings. Given the importance of learning and implementing self-directed strategies in the treatment of ME/CFS—such as “pacing,” managing energy throughout the day, maintaining good sleep hygiene, and using relaxation techniques—training programs are a crucial aspect of care for these patients. The goal of these strategies is to avoid PEM and enhance HRQoL [14]. Additionally, understanding the disease is crucial for the comprehensive care of the immediate environment, including family members and school personnel. Therefore, the objective of this project was to develop, implement, and evaluate modular group education programs for children and adolescents with ME/CFS, as well as for their parents, siblings, and teachers.

Nearly all children and adolescents with ME/CFS experience substantial limitations in their (school) activities and social interactions [6]. As a result, in-person group training sessions are not feasible for this patient group. Consequently, this project developed training sessions based on the ModuS concept for digital implementation. Short online sessions provide these individuals with the opportunity to participate, even if leaving the house or bed is not possible. This approach allows them to receive education and connect with others who are affected. Eliminating the need for travel to and from the program venue is an additional advantage for this group. As parents are limited in their time resources due to caring for affected children and adolescents, handling the bureaucratic aspects associated with the illness, and siblings also have time constraints, online formats offer a viable option for participating in education programs. In the professional realm, online continuing education has become increasingly available and valued, particularly during the COVID-19 pandemic [22]. For school staff, online training is beneficial due to its accessibility and time efficiency.

The study design presented describes the development, implementation, and planned evaluation of an online training model for individuals with ME/CFS up to the age of 20 years, as well as their parents, siblings, and school staff. This model is part of a new interprofessional care approach.

## Methods

### Study Design

This is a prospective study without a control group and will be evaluated using both qualitative and quantitative analyses. The objective of this study is to conceptualize, implement, and evaluate online education programs based on ModuS for adolescents with ME/CFS, their parents, siblings, and school staff. In phase I, the online programs were developed by a multidisciplinary team, piloted, and revised. Phase II involved the implementation of the finalized programs. In phase III, the programs will be evaluated with the primary objective of investigating participant acceptance. This will be assessed by measuring attendance frequency and responses to the question “Would you recommend the program to others?” in the online education programs. Additional objectives include investigating participants’ satisfaction with the programs and their assessment of the program’s completeness. This will be evaluated using quantitative questionnaires. Further, interviews with the individual groups participating in the programs will qualitatively analyze themes of individual expectations and benefits. In phase IV, disease knowledge gain, changes in HRQoL, and overall satisfaction will be investigated within a larger cohort.

### Development of the Training Program (Phase I)

#### Patient Training

**Figure 1:**
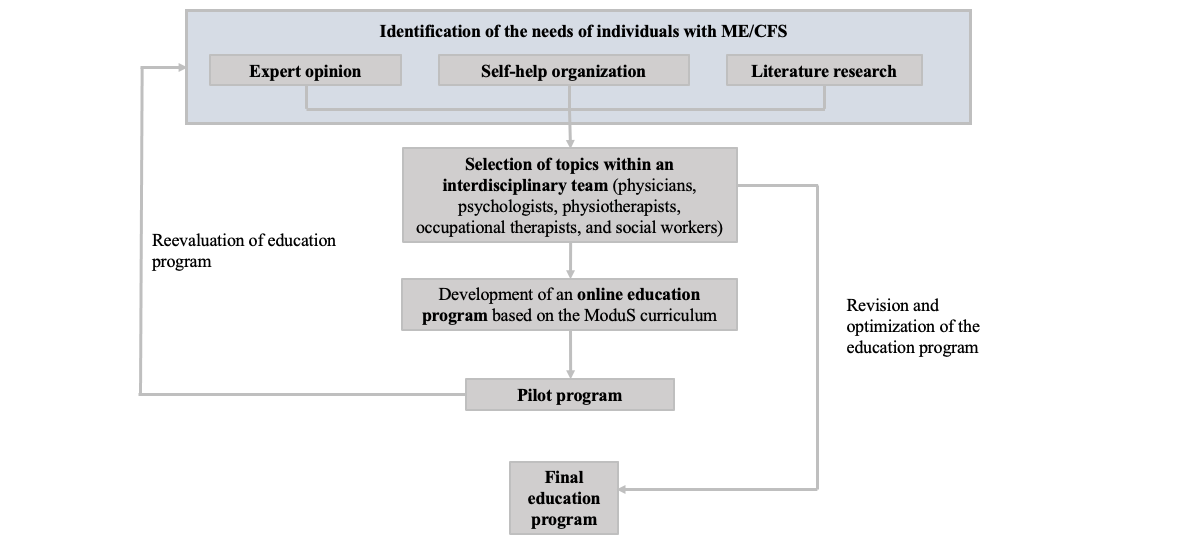
Process of developing the patient education program for affected children and adolescents. ME/CFS: myalgic encephalomyelitis/chronic fatigue syndrome.

In phase I of the education program development, the needs of individuals with ME/CFS were identified (Figure 1). This was achieved through consultations with ME/CFS experts and a literature review on the etiology, symptoms, and both pharmaceutical and conservative treatment options for ME/CFS in children and adolescents. The review was conducted using relevant databases such as PubMed, MEDLINE, and Cochrane Libraries. Additionally, input was gathered from patient organizations, which provided insights into the most common issues and challenges faced by affected individuals. Relevant topics were discussed within an interprofessional team, including members with ME/CFS from patient organizations, experienced physicians, psychologists, physiotherapists, and occupational therapists. Based on these discussions, the content deemed relevant to the affected individuals was selected.

The foundational structure of the training was based on the modules of the ModuS concept [20], with each training unit having a maximum duration of 45 minutes. This was complemented by customizable pre- and postprogram materials, considering the reduced endurance of patients with ME/CFS. The concept was pilot-tested in an initial program block from March to May 2023. Following this, the concept was reevaluated in collaboration with participants, experts, and patient organizations. Based on the feedback received, the education program was revised by the team, focusing on aspects such as flow, time allocation, and training materials. The final version of the education program was then developed.

#### Parent, Sibling, and School Staff Training

In addition to the online education program for affected children and adolescents, a slightly modified program concept was developed for parent training. The development process for this program was identical to that of the adolescent and young adult training, including collaboration with the interdisciplinary team and patient organizations. An education program specifically designed for minor siblings of affected individuals was also developed. This program was conceptualized in consultation with individuals affected by the condition and experienced health care providers. Another target audience for the online education program includes teachers and school staff. Given their significant role in the adolescents’ environment, it is crucial to inform schools about the condition and work together to determine how to support affected individuals in the educational setting, enabling them to continue participating in education and school life. The development process for this program was identical to that used for the training program for siblings.

After being piloted from March to May 2023, the programs were reevaluated, revised, and optimized.

### Recruitment and Implementation (Phase II)

#### Recruitment

Our specialized tertiary care center for young people with ME/CFS recruited participants up to the age of 20 years for online educational training after confirming the diagnosis during a short inpatient stay. Parents were recruited through the selected participants. Siblings and school personnel were informed about the training opportunities via patient organizations, a website, and the affected individuals themselves.

#### Implementation

Each training session was conducted by 2 professionals, with at least one having completed the patient trainer certification at the basic competence level according to KomPaS. This certification includes knowledge and skills in patient education as well as didactic tools. A maximum of 14 participants was allowed per training block.

### Evaluation (Phases III and IV)

#### Instruments

The selected measurement instruments include validated scores as well as self-developed questionnaires. Epidemiological data collected are gender, age, and type of school. Educational attainments are also queried from parents and school staff.

The evaluation forms for the different training sessions were developed based on the patient training assessment forms by Meng et al [23] and adapted to the program. These forms include items for assessing the content of the entire education program, as well as specific sessions, the format of the program, group dynamics, and practical usefulness in daily life. They investigate the satisfaction and completeness of the education programs. Response options are provided on a scale of 1=very good to 6=not poor, similar to a grading system. Additionally, 2 open-ended questions are included.

For assessing the perceived burden of the disease and HRQoL, patients complete a questionnaire with selected International Classification of Functioning, Disability and Health (ICF) items [24], the 36-item Short Form Health Survey (SF-36) [24,25], and the Bell score [26]. Parents complete the SF-36 and Bell score. Siblings complete the Large Analysis and Review of European Housing and Health Status (LARES) [27] and the Sibling Perception Questionnaire (SPQ) [28].

The selected ICF items cover domains such as learning, communication, mobility, self-care, household tasks, interpersonal relationships, school/work, and leisure, according to the ICF [24]. These items were selected through consensus among the interdisciplinary team, ensuring a comprehensive representation of important aspects for the participants.

The SF-36 covers 8 areas: physical functioning, bodily pain, general health perceptions, physical role functioning, emotional role functioning, mental health, vitality, and social functioning [25].

The SPQ includes questions about interpersonal and intrapersonal difficulties, as well as open communication with other caregivers about the disease. Responses are provided on a 4-point Likert scale (0=never to 4=always).

The LARES questionnaire is a screening tool for detecting distress in healthy siblings. It covers topics such as sibling relationships, social integration, family burden, school competence, and knowledge about the illness [27]. Response options are provided on a 4-point Likert scale (0=never/not at all to 4=always/very strongly).

For phase IV, the questionnaires will be adapted and shortened for the rollout.

#### Procedure

**Figure 2:**
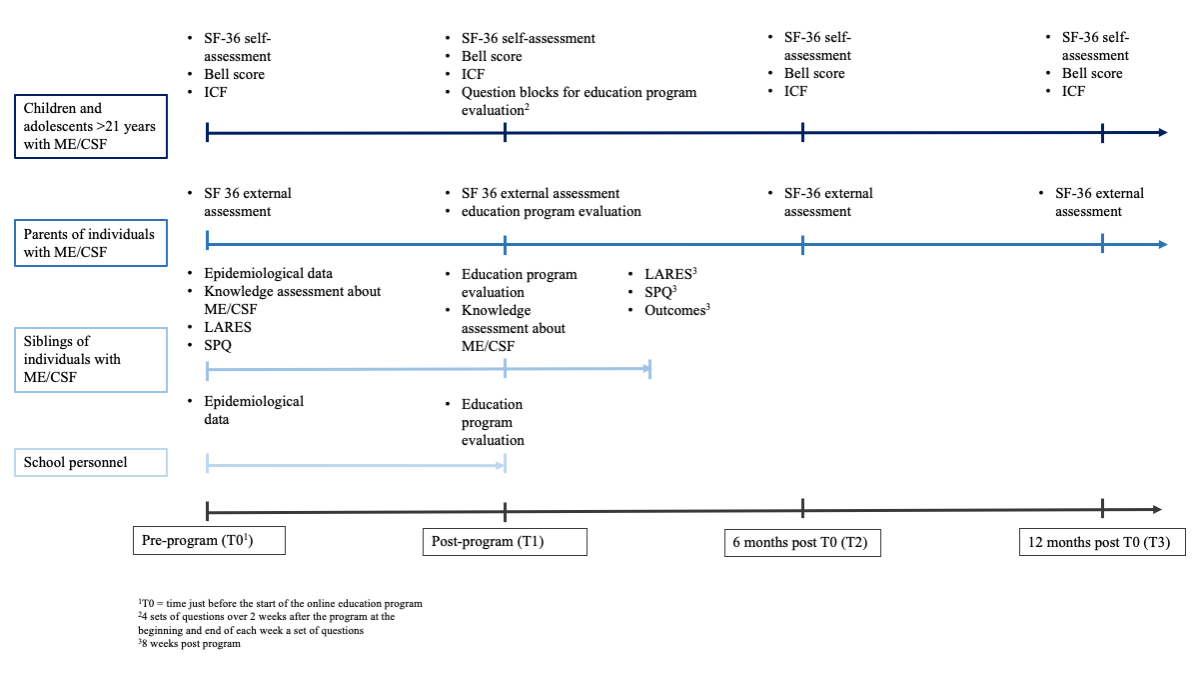
Overview of the evaluation of the study. ICF: International Classification of Functioning, Disability and Health; LARES: Large Analysis and Review of European Housing and Health Status; ME/CFS: myalgic encephalomyelitis/chronic fatigue syndrome; SF-36: 36-item Short Form Health Survey; SPQ: Sibling Perception Questionnaire.

The evaluation will involve a quantitative analysis of the questionnaires and a qualitative analysis of the interviews (Figure 2).

During recruitment, participants were invited to consent to receiving quantitative questionnaires. Participants with ME/CFS who agreed were surveyed at baseline, before the start of the online education program (T0). They were assessed regarding their participation, HRQoL, and perceived burden using selected ICF participation goals, the SF-36 questionnaire, and the Bell score. This baseline assessment aimed to evaluate the feasibility of the instruments for phase IV. The same questionnaires are administered immediately after the training (T1) and again at 6 and 12 months after the training (T2 and T3). Given the participants’ low energy levels, a total of 4 questionnaire blocks containing 10 questions each will be sent over 2 weeks, starting at the end of the training (T1). Responses will be recorded and submitted using a mobile device and will be qualitatively analyzed after transcription. Parents will complete the SF-36 questionnaire at baseline (T0) and immediately after the training (T1) for external assessment. Additionally, at T1, participants will receive questionnaires related to training evaluation. School staff will be surveyed at baseline (T0) for epidemiological data and will receive a training evaluation questionnaire immediately after the training (T1). Siblings will be surveyed at baseline (T0) for their epidemiological data and knowledge about the ME/CFS condition. Their daily burden due to their sibling’s chronic disease will also be assessed using the LARES and SPQ questionnaires. Following the training (T1), questionnaires are sent to evaluate the training and assess participants’ knowledge about the condition. Additionally, 8 weeks after completing the training, the LARES and SPQ questionnaires are administered, along with an assessment of the training’s impact on participants’ satisfaction.

For further qualitative investigation of the training program, semistructured interview guides were developed for school personnel, siblings, and parents by a multidisciplinary team. These interviews will be conducted after the completion of the programs. The guides aim to capture the impact of the education program on participants, as well as their individual expectations and perceived benefits.

In phase IV, the rollout of the program is planned for 150 children and their caregivers. The evaluation will focus on disease knowledge, HRQoL, life satisfaction, family burden, and longitudinal assessment at the following time points: T-1 (registration), T0 (before the start), T1 (after completion), T2 (6 months after completion), and T3 (12 months after completion).

#### Data Processing

The data platform REDCap (Research Electronic Data Capture; Vanderbilt University) is used for sending out questionnaires and storing data. A separate database was created for each survey, containing the corresponding questionnaires. If training participants agreed to participate and provided their email addresses, their information was entered into the databases. Questionnaires are sent as links in emails at the designated time points. Each study participant is assigned an ID, which is linked to their corresponding email address in a separate document. This document is stored in a manner that allows only study staff with access to trace back the identity. Linking will only occur for combining the SF-36 self-assessment and external assessment by the participants and their parents. All other questionnaires will be stored and analyzed anonymously. In cases of missing or incomplete questionnaire responses, participants will receive reminder emails after 1 week. Audio files containing responses to questionnaire sections can be uploaded and stored using the clinic’s internal data exchange program, “fex.” Transcription backups will be stored exclusively on the clinic’s secure server. The analysis will be conducted using pseudonymized data only.

### Statistical Analysis

The study follows an exploratory approach. The goal is to obtain responses from 30 participants for the quantitative surveys, which is projected to provide sufficient statistical power to assess acceptability. This will be measured by the variable “Would you recommend the program to others?” on a scale from 1 to 6, with responses dichotomized as follows: 1-2=high acceptance and 3-6=low acceptance. Based on the EduKids study, which involved staff training on diabetes [29], a comparable proportion of 87% with high acceptance is assumed. A sample size of 30 participants is deemed sufficient to estimate the proportion of individuals with high acceptance of the training at 87%, with a precision of 12% (half the width of the 95% CI according to the Wilson score). The quantitative questionnaire data will be analyzed using SPSS software (version 29.0.1.1; IBM Corp.). Questionnaires from different training programs will be analyzed separately.

Epidemiological data of participants, as well as their satisfaction, perceived usefulness, completeness of the modules, knowledge gain, and acceptance (in terms of attendance rates) across different training programs, will be represented through descriptive analyses, including means and SDs. For categorical variables, such as gender and severity of disease, results will be presented using both relative and absolute frequencies. The perceived burden of the disease will be assessed through various specific questionnaires (SF-36, Bell, LARES, and SPQ) and analyzed using descriptive statistics. Changes in these variables before and after the programs will be analyzed using statistical tests for dependent samples, such as the paired 2-tailed *t* test.

A significance level of *P*≤0.05 will be assumed for all procedures.

### Qualitative Analysis

In the qualitative analysis of interviews, an exploratory analysis will focus on themes related to individual benefits and satisfaction. Achieving saturation is not a specific target.

Interviews with parents, siblings, and school staff will be conducted using semistructured interview guides. The questions will be developed through collaboration between interdisciplinary teams and external researchers with expertise in qualitative analysis, through multiple discussion rounds. As a result of the dispersed geographical distribution of participants, all interviews will be conducted online and recorded. The recordings will be pseudonymized and transcribed verbatim. The analysis will be carried out using MAXQDA software (VERBI Software). Category creation will involve both deductive approaches, based on themes outlined in the interview guide, and inductive approaches, derived from the data. The qualitative content analysis method by Kuckartz [30] will be used as a guideline. After an initial analysis of the material, all transcripts will be independently analyzed again by a second person, with categories formed using the same process. The analyses and categories from both iterations will be discussed within the team, and the material will be reanalyzed based on the revised categories. The analyses were regularly discussed and debated within an interdisciplinary team consisting of physicians, psychologists, physiotherapists, social workers, and occupational therapists. Upon reaching a consensus, a category handbook will be created, and the results will be presented using hierarchical code-subcode models and word clouds.

### Ethical Considerations

The study was conducted in accordance with the Declaration of Helsinki. The Ethics Committee of the University Hospital of the Technical University of Munich approved the BAYNET FOR ME/CFS study (approval number 2023-1-S-KH). Additionally, the Ethics Committee of Julius-Maximilians-University approved the conduct of the training evaluation on August 11, 2023 (approval number 71/23). Written informed consent will be obtained from participants or, for children under 18 years, from a parent or legal guardian after recruitment. Participation is voluntary, and there will be no consequences for not participating or completing the study. The confidentiality and privacy of participants’ information will be protected by deidentifying the data and using a secure, encrypted, and password-protected database for storage.

## Results

### Piloting

All programs were pilot-tested in an initial phase from March to May 2023, as part of phase I. Participants were recruited through a patient organization and advertisements on the institute’s website. In total, 8 patients and their parents, 5 siblings, and 59 school staff took part in the piloting. Participants who participated in the pilot testing were not eligible to participate in the finalized online education programs.

### Finalized Online Education Programs

In total, 4 programs were developed for young individuals with ME/CFS, their parents, siblings, and school staff. In the final version of the online education program for young individuals with ME/CFS, the generic modules on introduction and disease management were placed at the beginning and end of the program, following the ModuS curriculum (Table 1). As a result of the limited endurance of patients, the content was condensed and shortened. Each unit included brief practical sequences alongside theoretical input and group discussions. Theoretical content was regularly reviewed with the affected individuals, individual barriers were identified, and collaborative solutions were developed within the group. Presentations for each program unit were created in consultation with patient organizations and included a comic story. Before the education program sessions, participants can familiarize themselves with the topics through these comics or short videos/podcasts, along with small preparatory tasks. For the postsession phase, each unit’s content is summarized in an age-appropriate comic, which will be provided to participants for reflection and as a reminder.

**Table 1:** Overview of units of the online ME/CFS^a^ education program for children and adolescents with ME/CFS based on the ModuS curriculum^b^.

ModuS modules^c^	Description of modules	Program units	Content	Methods/materials	Profession of trainer
0 + I + II^b,d^	Introduction and motivating education about the illness, treatment, and prognosis	1. Introduction	Introduction, team presentation, group formation, disease education, symptoms, and introduction to pacing diary	Training materials, group discussions, and surveys	Physician
III + IV^e^	Skills and motivation for low-symptom intervals and skills for regulating and avoiding acute crises: emergency management	2-3. Breathing and relaxation exercises and strategies	Reflection on previously known relaxation strategies, importance and benefits of relaxation techniques, and teaching of relaxation and breathing exercises (diaphragmatic breathing, body scan, progressive muscle relaxation, guided imagery)	Training materials, breathing exercises, and surveys	Physiotherapist
II + IV^e^	Motivating education about the illness, treatment, and prognosis and skills for regulating and avoiding acute crises: emergency management	4-5. PEM^f^/crash	Reflection on individual stressors, definition of PEM, strategies for managing PEM, and exercise for applying pacing to prevent PEM	Training materials, group discussions, and surveys	Occupational therapist
III + IV^e^	Skills and motivation for low-symptom intervals and skills for regulating and avoiding acute crises: emergency management	6-7. Pacing	Definition of pacing, pacing rules, energy-saving techniques, and development of individual pacing strategies	Training materials, surveys, and group discussions	Occupational therapist
III + IV^e^	Skills and motivation for low-symptom intervals and skills for regulating and avoiding acute crises: emergency management	8-9. Additional therapies	Current medication treatment options, main pillars of ME/CFS therapy, treatment options for sleep disorders, circulatory issues, pain, and brain fog	Training materials, surveys, and group discussions	Physician
V^d^	Disease management within the family system	10-11. Disease management	Current emotional energy drains, teaching emotion regulation strategies, and practice for recognizing one’s own emotional capacity	Training materials, surveys, individual work, and group discussions	Psychologist
VI^d^	Conclusion	12. Open discussion	Formulating personal goals for the future, feedback opportunities, and conclusion	Surveys and group discussions	Physician

^a^ME/CFS: myalgic encephalomyelitis/chronic fatigue syndrome.

^b^The unit includes topics from both cross-disease and disease-specific modules.

^c^All modules are conducted by 2 trainers, with the lead professional for each session indicated in the table. Either a physician or a psychologist participates in every session to ensure the continuity of the program.

^d^Generic modules.

^e^Diagnosis-specific modules.

^f^PEM: postexertional malaise.

All online program units for the affected individuals were conducted in the early afternoon and were scheduled for 45 minutes. Participants had the option to end the session early if they felt exhausted. During the project, 2 training blocks, each consisting of 12 sessions, were conducted, involving a total of 24 participants. Each training unit was led by 2 trainers with at least one certified as a ModuS patient trainer. The objective was to empower the affected individuals to become “experts” in managing their own condition.

For the parents’ online education program, in addition to information about ME/CFS, care strategies, and therapy, social, legal, and educational topics were included. The units were tailored to the needs of parents (Table 2). The focus was on current treatment approaches, particularly pacing, as well as relaxation and breathing techniques, which are central to ME/CFS therapy. Parents were given the opportunity to address emotionally distressing experiences related to ME/CFS. The parental program consisted of 4 evening sessions, each conducted online and lasting 1.5 hours. Similar to the program for affected patients, it was designed to support and inform parents in managing their child’s condition.

In addition to providing information about the disease, the siblings’ program focused on the experiences of siblings and offered them an opportunity to share their thoughts. The program was based on the ModuS sibling workshop but was condensed to fit an online format within 1 afternoon. For school staff, an online education program was developed to provide information about ME/CFS and its symptoms, as well as strategies for supporting affected individuals within the educational setting.

Both the sibling and school staff training sessions consisted of 90-minute online meetings held in the evening via a conventional online platform.

**Table 2:** Overview of modules of the online ME/CFS^a^ education program for parents of children and adolescents with ME/CFS based on the ModuS curriculum^b^.

ModuS module	Module description	Program units	Content	Methods/materials	Profession of trainer
0 +I + II + V^b,c^	Introduction and motivational education on illness, treatment, prognosis, and disease management within the family system	1. Disease pathogenesis and coping	Introduction, team introduction and training structure, explanation of ME/CFS pathophysiology and symptoms, and presentation of the 5-phase model of illness coping and coping strategies	Training materials, small group activities, and surveys	Physicians and psychologists
III + IV^d^	Skills and motivation for low-symptom intervals and skills for regulating and avoiding acute crises: emergency management	2. Breathing and relaxation exercises and strategies and pacing	Importance and benefits of relaxation techniques as well as teaching relaxation and breathing exercises (diaphragmatic breathing, body scan, muscle relaxation, and guided imagery)	Training materials, breathing exercises, and survey	Physiotherapists and occupational therapists
II + IV + V^b,c^	Motivational education about the illness and skills for regulating and avoiding acute crises: emergency management and disease management within the family system	3. Therapy options and management of disease	Presentation of conservative treatment measures for the most common symptoms of ME/CFS, reflection on individual family challenges, effects of chronic stress, guidance for dealing with stress and promoting emotional stability, and reflection on challenges and strategies for dealing with the social environment	Training materials, surveys, and group work	Physicians and psychologists
III + IV^d^	Skills and motivation for low-symptom intervals and skills for regulating and avoiding acute crises: emergency management	4. Social legal aspects and school	Information on social legal entitlements and presentation of various educational participation options	Training materials	Social workers

^a^ME/CFS: myalgic encephalomyelitis/chronic fatigue syndrome.

^b^The unit includes topics from both cross-diseases and disease-specific modules.

^c^Generic modules.

^d^Diagnosis-specific modules.

### Implementation of the Education Programs

The goal of recruiting 30 patients was not achieved, as some patients had previously participated in in-hospital training and declined to join the online program. Recruitment of siblings was also unexpectedly low despite advertising through various channels. Between January 2024 and June 2024, the study successfully recruited 24 young patients with ME/CFS and their parents, along with 8 siblings and 51 school staff. The participants agreed to take part in both the programs and their evaluation. The online education programs, which included 2 program blocks for patients and parents and 2-3 blocks for siblings and school staff, commenced in May 2023 and concluded by the end of October 2023. All participants were surveyed at baseline (T0) before the programs began, and ongoing evaluations will continue through the end of 2024. Data analysis will begin after the completion of the programs and is anticipated to be finalized by the end of 2024.

During recruitment, participants had the option to agree to interviews. From those who consented, 3 participants attending school and 3 participants not attending school were selected for interviews after completing the program, along with parents who agreed to participate in interviews. Additionally, for siblings and school staff, 6 individuals who consented to interviews were randomly selected after the program concluded.

In phase IV, the rollout of the program is planned for 2025-2026, targeting 150 children and their caretakers. This phase will focus on investigating disease knowledge, HRQoL, life satisfaction, family burden, and conducting a longitudinal evaluation.

## Discussion

### Overview

Currently, there are only a few specific and regionally limited health care options available for children and adolescents with ME/CFS. Educational programs for both patients and their social environment represent a crucial step toward providing comprehensive care for these individuals. This study aims to design, implement, and evaluate an online education program for children and adolescents with ME/CFS, as well as for their social environment, including parents, siblings, and school personnel. The training programs are developed based on the ModuS training concept [20] and aim to empower participants in the self-management of ME/CFS. They provide a comprehensive understanding of the disease and promote exchange among those affected.

### Primary Findings

In contrast to typical standardized education concepts, the ME/CFS education programs for all target groups were designed as online offerings. For patients, these online sessions allowed participation even when leaving the house or bed is not possible. By offering the training program online, adolescents could receive disease-specific education and connect with other affected individuals. Additionally, the program sessions could be attended from various positions, such as lying down, which minimizes physical exertion. Participants could also shorten their involvement if their energy levels declined during the session. The elimination of the need to commute further alleviated the burden on this group. Most electronic devices, such as PCs, laptops, and mobile phones, equipped with cameras, enabled virtual introductions and direct interactions among participants. Hence, the core advantage of this patient education program—interaction and networking—remained intact in the digital format.

For parents, who are often constrained by the care of affected children and the bureaucratic demands associated with the disease, the online format provides a practical solution. It eliminates the need for time-consuming travel and offers flexibility, allowing them to participate from various locations. Similarly, for siblings, the online format reduces barriers to participation, enabling them to connect easily and participate without needing parental support for mobility.

In the professional realm, online events have become increasingly common, particularly due to the COVID-19 pandemic, and are now widely offered and appreciated [22]. Consequently, the education program for school staff was also designed as online sessions to maximize reach, leveraging the accessibility and time efficiency of this format.

The design of the education programs accounted for the individual needs of each participant beyond the digital format. Given that ME/CFS is often associated with significant fatigue and reduced concentration spans [14], the education program units for patients were kept brief and included minimally taxing practical exercises. Therefore, each unit was limited to 45 minutes. To mitigate the risk of PEM associated with ME/CFS [6], participants were encouraged to assess their energy levels at the start, midway, and end of each session. A short break was provided midway to prevent overexertion. Participants were also informed that they could leave the session early if they reported low energy levels. Trainers did not exclude any participants due to low energy, as managing one’s own resources was a central learning objective of the training.

Given the time limitations of each unit, preparatory and follow-up materials, including podcasts, were developed for each training session. This approach allows participants to review these materials at their convenience, according to their energy levels. To enhance comprehension for children and adolescents, materials were presented visually as comics, avoiding lengthy text passages. Videos and podcasts were incorporated to support multisensory learning and facilitate information absorption.

The ModuS curriculum was initially developed for managing rare or chronic diseases [20]. Its positive impact on disease management and HRQoL has been consistently demonstrated in various studies [16,31].

The modular structure of the ModuS concept, which provides predefined topics with a high degree of flexibility, allows for the customization of content to meet the specific needs of individuals with ME/CFS and their social environment. Efforts were made to select content that is most relevant to patients with ME/CFS, their parents, siblings, and school staff.

Consistent with the ModuS concept, the training for patients extended beyond medically relevant subjects to encompass psychosocial themes that are common across chronic diseases [20].

The training sessions for parents were designed to cover essential aspects such as navigating their child’s illness, communicating it in a child-friendly manner, and accessing specialized medical support [6]. Given that the disease imposes various burdens on parents [32,33], which can significantly affect daily family life, the training aimed to address these challenges comprehensively. Therefore, the program not only addressed the origins and treatment of the disease but also dedicated an entire session to disease management. Recognizing that parents of patients with ME/CFS often lack information about social and legal aspects [32], these topics were incorporated into the program through collaboration with social pedagogues. This approach aimed to provide a comprehensive understanding of the various dimensions of managing ME/CFS and its impact on families.

The content for the sibling training was selected based on the unique challenges faced by siblings of children with chronic illnesses. These illnesses can place burdens on siblings as well [34], potentially leading to feelings of shame, jealousy, and anxiety [6]. Additionally, there may be a redistribution of resources, such as time spent with parents, increased family stress, and social challenges [

35]. This training primarily focuses on providing a child-friendly explanation of the illness. Additionally, it aims to foster understanding of the disease and empathy for the affected family member. The sibling training emphasizes facilitating interaction among affected siblings and creating a platform for them to express their needs, questions, uncertainties, and fears.

### Limitations

This study has several limitations. First, the number of participants is limited, and their participation in both the program and the evaluation is voluntary. This may result in a disproportionate representation of less severely affected patients, who might be more capable of taking part in the program and evaluation, potentially biasing the responses. Additionally, this could lead to higher acceptance rates compared with more severely affected patients, who may be less able to participate due to the severity of their condition. However, we plan to address this by conducting interviews and qualitative analyses that include patients with varying degrees of disease burden, incorporating these results into the overall assessment. Second, patients were recruited from only 1 center, which may disproportionately represent individuals from a specific region. Nevertheless, this is the only center in Germany for young patients with ME/CFS, ensuring accurate diagnosis and adherence to the inclusion criteria. Lastly, the evaluation of the program is conducted immediately after its completion, so no conclusions can be drawn about the long-term effects of the programs in phases I-III.

### Conclusions

This study protocol outlines the study design, goals, conceptualization, and implementation of newly developed online training programs for individuals up to the age of 20 years with ME/CFS, as well as their parents, siblings, and school staff. The primary aim of the training is to enhance disease knowledge and empower affected individuals to manage their own condition. Individuals with ME/CFS often face significant limitations in social participation due to restricted mobility. Online training programs not only support affected individuals in managing their disease but also foster social participation through interactions with others who are similarly affected.

However, the impact of the disease extends to the social environment, including parents, siblings, and school personnel. There is a notable knowledge gap in these groups, which the training programs, as part of an interprofessional care model, aim to address. The newly established interdisciplinary training program, based on the ModuS principle for ME/CFS, can make a valuable contribution to raising awareness about the condition and expanding regional coverage, thereby significantly enhancing the care of children and adolescents with ME/CFS.

Following a positive evaluation, plans are underway to establish train-the-trainer programs to facilitate the widespread implementation of ME/CFS training as part of a standardized care model for affected children and adolescents. The high demand for the pilot training programs underscores the need for such initiatives, not only among affected individuals but also among parents, school staff, and siblings. In the future, multiple training blocks are planned to be offered throughout the year, contributing to comprehensive care. These programs will be adjusted and improved based on the initial evaluations.

## Abbreviations

EUROMENE: European Network on ME/CFS
HRQoL: health-related quality of life
ICF: International Classification of Functioning, Disability and Health
KomPaS: Competence Network for Patient Education
LARES: Large Analysis and Review of European Housing and Health Status
ME/CFS: myalgic encephalomyelitis/chronic fatigue syndrome
PEM: postexertional malaise
REDCap: Research Electronic Data Capture
SF-36: 36-item Short Form Health Survey
SPQ: Sibling Perception Questionnaire

## Data Availability

The data sets analyzed during this study are available from the corresponding author on reasonable request.
